# A gut instinct for childhood leukemia prevention: microbiome-targeting recommendations aimed at parents and caregivers

**DOI:** 10.3389/fpubh.2024.1445113

**Published:** 2025-01-13

**Authors:** Ersen Kameri, Vera Helena Jepsen, Pawel Stachura, Nadine Rüchel, Rigveda Bhave, Leticia Benitez, Fatima Crispi, Eduard Gratacos, Nico Dragano, Stefan Janssen, Arndt Borkhardt, Aleksandra Pandyra, Gesine Kögler, Ute Fischer

**Affiliations:** ^1^Department of Pediatric Oncology, Hematology and Clinical Immunology, Medical Faculty, Centre of Child and Adolescent Health, Heinrich-Heine-University, Düsseldorf, Germany; ^2^Cancer Prevention-Graduate School, German Cancer Research Center (DKFZ), Heidelberg, Germany; ^3^Institute of Transplantation Diagnostics and Cell Therapeutics, Medical Faculty, Heinrich-Heine-University, Düsseldorf, Germany; ^4^Department of Molecular Medicine II, Medical Faculty, Heinrich-Heine-University, Düsseldorf, Germany; ^5^BCNatal, Fetal Medicine Research Center (Hospital Clinic and Hospital Sant Joan de Déu), University of Barcelona, Institut d'Investigacions Biomédiques August Pi i Sunyer (IDIBAPS), Barcelona, Spain; ^6^Institute of Medical Sociology, Centre for Health and Society, Medical Faculty, Heinrich-Heine-University, Düsseldorf, Germany; ^7^Algorithmic Bioinformatics, Department of Biology and Chemistry, Justus Liebig University Gießen, Gießen, Germany; ^8^German Cancer Consortium (DKTK), Partner Site Essen/Düsseldorf, Düsseldorf, Germany; ^9^Institute of Clinical Chemistry and Clinical Pharmacology, University Hospital Bonn, Bonn, Germany; ^10^German Centre for Infection Research (DZIF), Partner Site Bonn-Cologne, Bonn, Germany

**Keywords:** childhood leukemia, risk factors, gut microbiome, prevention, recommendations, public health

## Abstract

Childhood leukemia accounts for 30% of all pediatric cancer cases with acute lymphoblastic leukemia (ALL) being the most common subtype. Involvement of the gut microbiome in ALL development has recently garnered interest due to an increasing recognition of the key contribution the microbiome plays in maintaining the immune system's homeostatic balance. Commensal gut microbiota provide a first line of defense against different pathogens and gut microbiome immaturity has been implicated in ALL pathogenesis. Several environmental factors such as nutrition, mode of delivery, breastfeeding and, early social or livestock contacts are known to alter the composition of the gut microbiota. Variations in these factors influence the risk of childhood leukemia onset. This review aims to elucidate the risk factors influencing microbial composition in the context of childhood ALL. The link between gut microbiome diversity and childhood ALL offers the opportunity to develop risk-reducing strategies that can be communicated to a broad target population of (future) parents and caregivers for childhood leukemia prevention. Here, we summarize evidence on how promoting a diverse gut microbiome in newborns through simple measures such as increasing social contacts early in life may decrease the risk of developing ALL in these children later on.

## 1 Introduction

**Figure 1:**
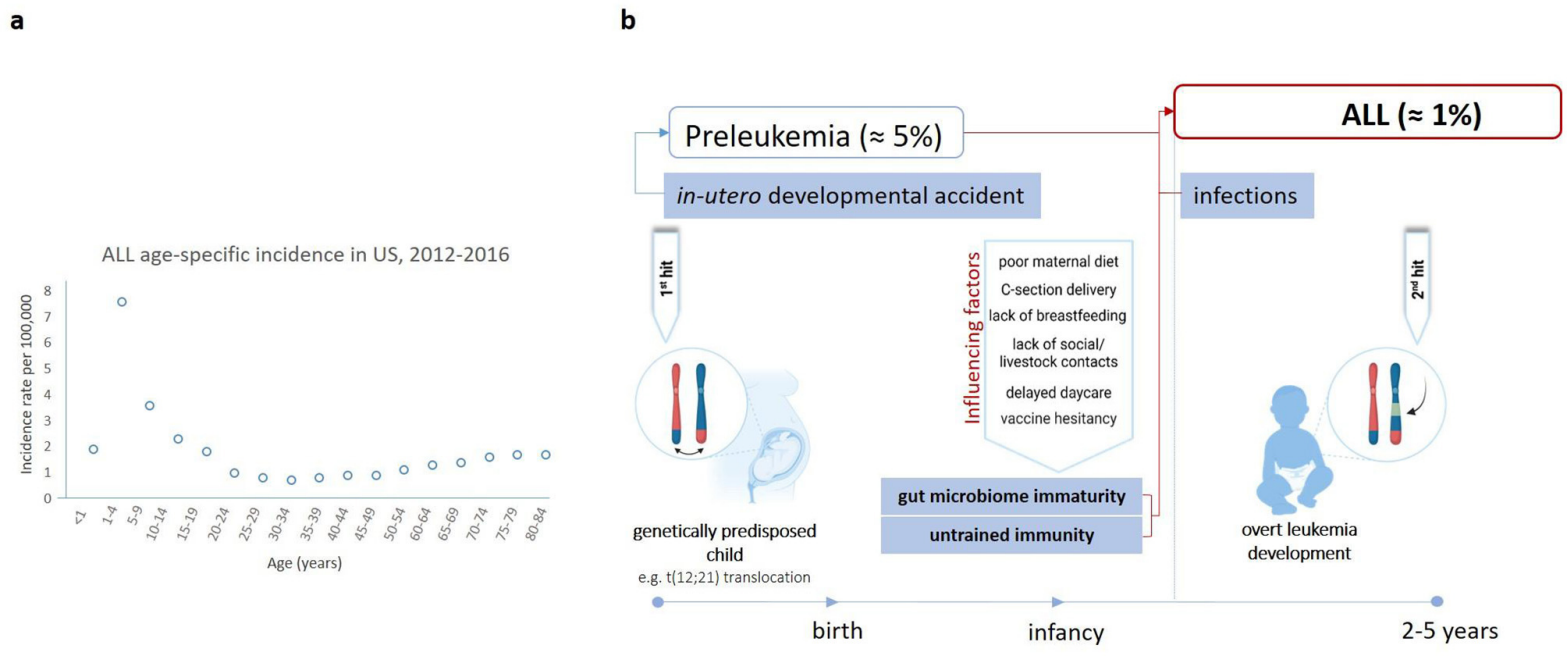
ALL age distribution and the “two hit and delayed infection model” of ALL pathogenesis. **(A)** ALL age distribution in the USA from 2012 to 2016, with a peak at 1–4 years. Incidence per 100,000 cases is shown (2). **(B)** The “two-hit theory” suggests that ALL occurs in a two-step process (3). The first one involves a genetic mutation, e.g., the *t*_(12,21)_ translocation, which arises *in-utero* predisposing the child to leukemia (~1–5% of healthy newborns) (9). However, the acquisition of additional mutations is crucial for leukemia onset (3). These mutations are primarily driven by oncogenic deletions occurring in ~1% of genetically predisposed children likely due to a dysregulated immune response upon exposure to one or more common infectious agents (3). This is especially critical for those children who were raised in an exaggerated hygienic environment, pointing to a potential association between the gut microbiome and ALL development (10). Hence, other factors affecting gut microbiome diversity, including maternal diet, mode of delivery, breastfeeding, vaccination, social, and livestock contacts are also thought to be implicated (10, 24). Figure was created on BioRender.com.

Cancer is the second most frequent cause of death among children in developed countries and acute lymphoblastic leukemia (ALL) is the most common subtype accounting for 30% of childhood cancer cases (1). ALL incidence peaks at 2–4 years of age (Figure 1A) and is increasing steadily (2). Around 80% of childhood ALL cases are characterized by proliferation of abnormal B-cell progenitors characterized as B-cell precursor ALL (BCP-ALL) (3). Although the 5-year survival rate of children with BCP-ALL has improved significantly, there are several adverse side effects associated with treatment (4). Today, there are about half a million childhood cancer survivors living in Europe and two-thirds of them suffer from acute and late treatment-related toxicities, accounting for a large proportion of deaths (4, 5). For instance, chemotherapy treatment for childhood ALL can affect all organs and cause acute and persistent organ damage (4). Common acute adverse effects include opportunistic infections, mucositis, neuropathy, thromboembolism, bone toxicities, endocrinopathies, hypersensitivity, pancreatitis, nephrotoxicity, thrombosis, and hyperlipidemia (3, 4). Long-term toxicity stemming from the treatment such as cognitive impairment, osteonecrosis, secondary cancers, infertility and, depression, can be severe and alter the socioeconomic participation of at least half of those affected (6). The increasing childhood cancer incidence in Europe highlights the need to shift the current paradigm from therapy to prevention (7). Preventive strategies could circumvent traumatic and toxic treatments, associated life-long health sequelae, and the experience of relapse or treatment resistant leukemia subtypes, which occur in about 20% of cases (8). In addition, adopting preventive strategies could significantly reduce the cumulative public health burden incurred by an increasing number of adult cancer survivors that suffer from the aftereffects of treatment and a decreased life quality (5).

**Figure 2:**
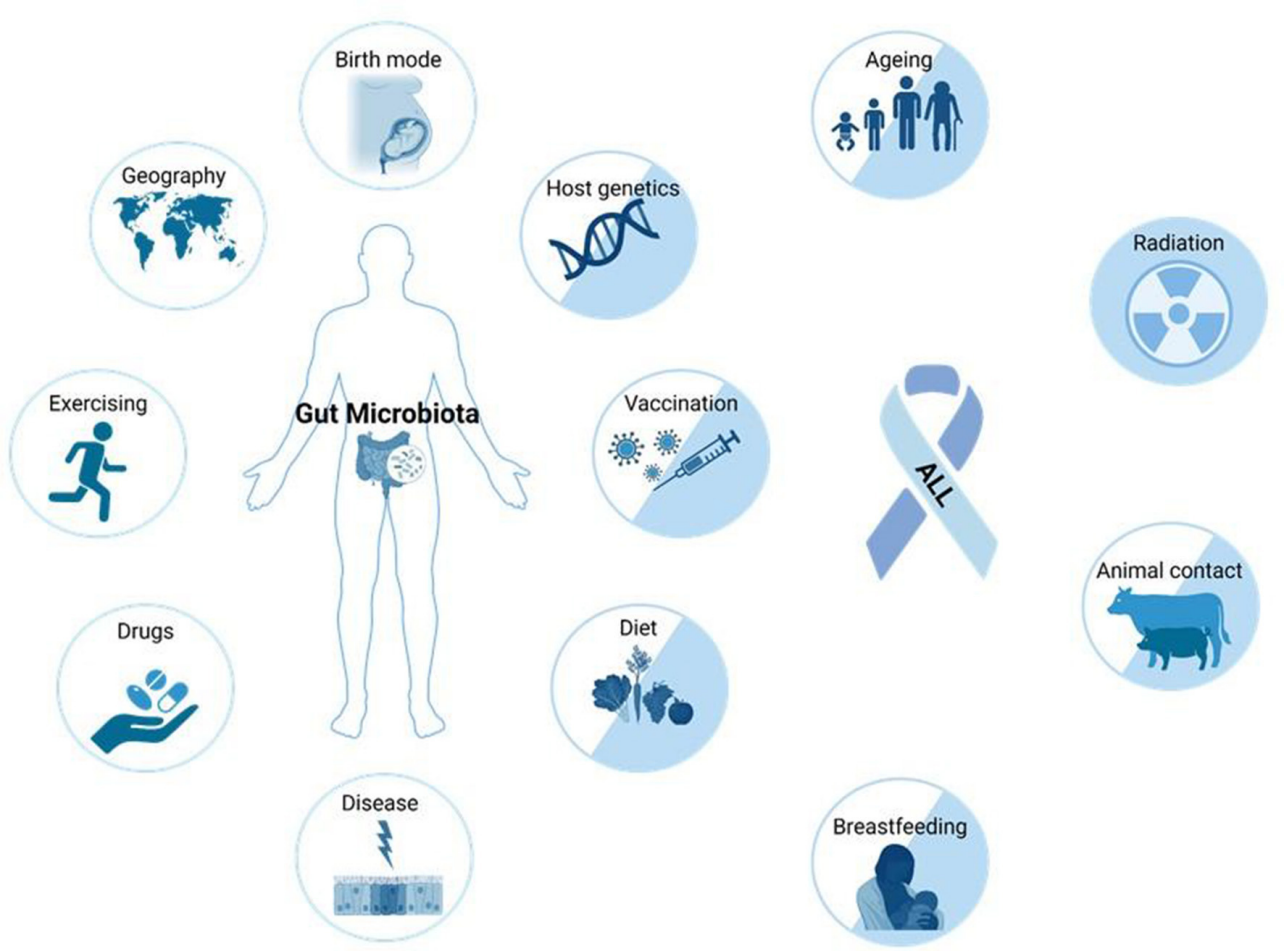
Factors that can influence the composition and function of the human gut microbiota, and alter ALL risk. Many external (drugs, exercise, geography, birth mode, vaccinations, diet, breastfeeding, and animal contact) and intrinsic factors (host genetics, disease, and aging) impact microbiome composition. The etiology of ALL is not yet fully understood, but causal connections to many factors accepted to influence the microbiome are also known to impact the risk of ALL development (factors represented by two-colored circles) (10). Figure was created on BioRender.com.

Childhood ALL is frequently triggered by genetic mutations somatically acquired before birth (Figure 1B) (3). The most common event is the translocation t(12,21)(p13;q22) generating the *ETV6::RUNX1* fusion gene (3). Secondary oncogenic gene alterations are necessary for overt leukemia and are likely driven by modifiable environmental and lifestyle factors, therefore making ALL in principle a preventable disease (9). Several factors known to increase the risk of developing ALL also have a strong impact on the composition of the gut microbiota (Figure 2).

Indeed, a potential causative involvement of the microbiome in the development of ALL is increasingly recognized (10). This is particularly relevant in the context of Greaves' delayed infection theory of childhood leukemia development proposing that exposure to common infectious agents may trigger leukemia in genetically predisposed children (11). The delayed infection theory highlights the role of typical common childhood infections, including respiratory or gastrointestinal pathogens, which might influence immune system dysregulation in genetically susceptible children (11). There are reports of BCP-ALL space-time clusters associated with different specific pathogens: adenovirus for 13 patients in the Fallon cluster, streptococcal fever for 8 same-school patients in the Niles cluster, and influenza A H1N1 swine flu virus for seven patients in the Milan cluster (12–15). Furthermore, Christoph et al. identified virus sequences corresponding to common human pathogens, such as *Anelloviridae, Herpesviridae*, and *Parvoviridae* family in 11 B-ALL cases analyzed by whole genome sequencing (16). In addition, a study in UK observed peaks of BCP-ALL ~6 months after seasonal influenza epidemics. According to Greaves (as illustrated in Figure 1B), if the exposure of a child's immune system to infectious triggers is delayed due to lack of social contacts or an exaggerated hygiene, the eventual immune response may be dysregulated and lead to the progression of pre-leukemic cells (3).

The gut microbiome and immune system co-develop during early infancy and childhood. During this period the gut microbiome plays a major role in shaping host's immunity (10). Commensal microbiota provide a first line of defense against different pathogens, and gut microbiome immaturity due to genetic or lifestyle factors results in untrained immunity which could promote the switch toward overt leukemia in genetically predisposed children (10). *In vivo* mouse models demonstrated that the lack of commensal microbiome alone could be sufficient to promote leukemia in genetically predisposed mice, simply by inducing a microbiome disruption via early-life antibiotic treatment (2). Infections combined with an altered microbiome composition may not only provide sufficient proliferative stimuli for pre-leukemic cells to induce the acquisition of secondary oncogenic driver mutations but also drive a pro-inflammatory and immunosuppressive hematopoietic niche supporting a leukemia-favorable microenvironment (3, 10).

**Figure 3:**
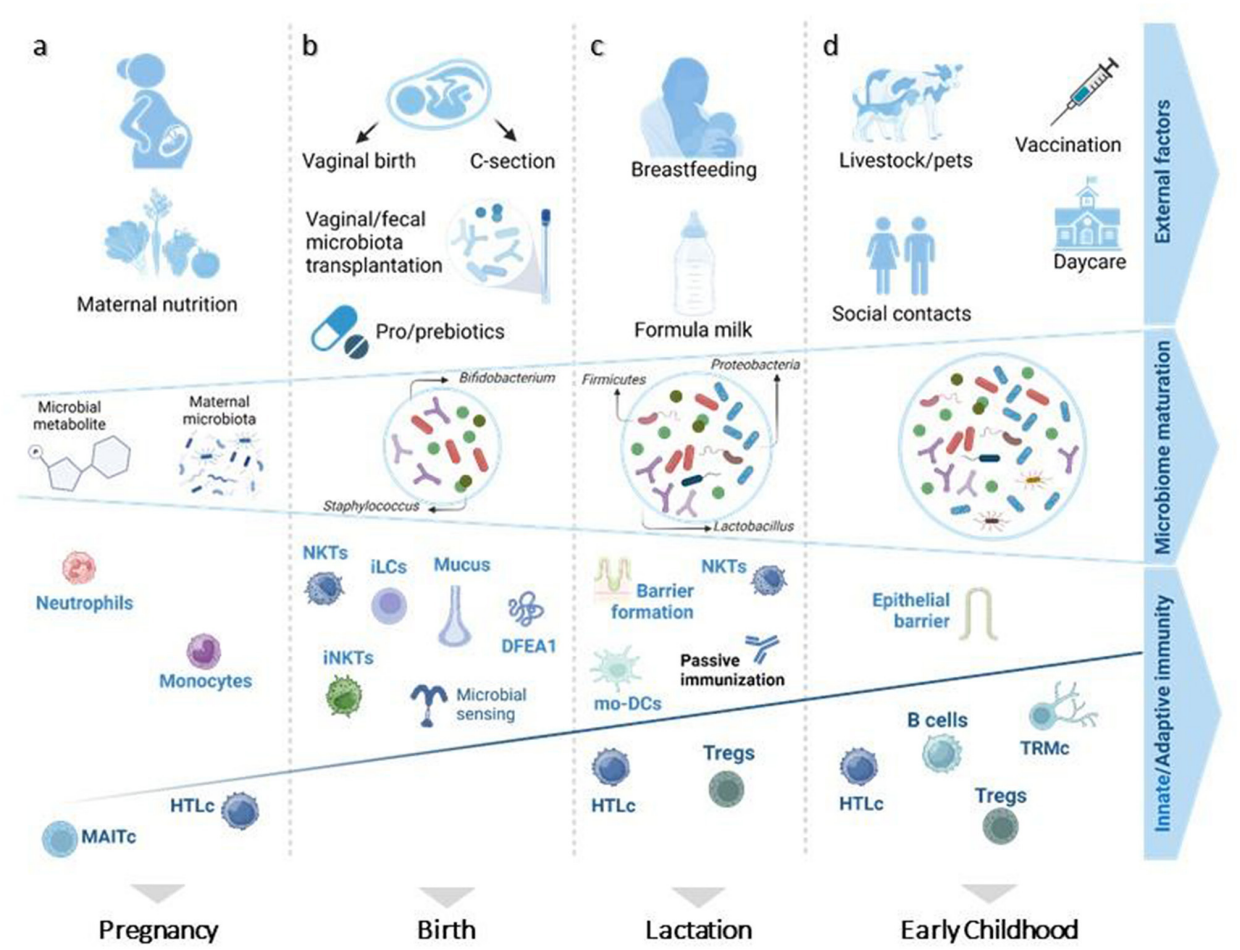
Prenatal and postnatal factors influencing microbiome diversity and immune system maturity of children. **(A)** *In-utero*, the presence of microbial-derived metabolites and maternal microbiota educates the immune system on how to confront postnatal microbes, affecting especially innate immune system cells (monocytes and neutrophils). **(B)** At birth, a neonate's immune system relies on maternal protection for its first encounter with living pathogens. **(C)** Breastfeeding plays an essential role in sustaining this protection by aiding in epithelial barrier formation and passive immunization. **(D)** Afterwards, increased microbiome diversity is followed by further evolvement of mucosal and adaptive immunity. Figure adapted from Kalbermatter et al. (17). MAITc, mucosal-associated invariant T-cells; HTLc, helper T lymphocyte cells; NKTs, natural killer T cells; iLCs, innate lymphoid cells; Tregs, regulatory T cells; iNKTs, invariant natural killer cells; DFEA1, alpha defensin 1; mo-DCs, monocyte-derived dendritic cells; TRMc, tissue -resident memory T cells. Figure was created on BioRender.com.

The gut microbiome and the immune system develop simultaneously starting *in-utero*. Different prenatal and postnatal factors influence this process (Figure 3) (17). During pregnancy, placental transmission of bacterial-derived metabolites originating from maternal diet and microbiota initiate the priming and development of the immune system, emphasizing the importance of a healthy maternal nutrition in preventing ALL (17, 18). Although essential components of both innate and adaptive immunity already develop at the prenatal stage, they predominantly evolve after birth alongside with the diversification of the microbiome (17, 19). At birth, the mode of delivery directly determines the most abundant type of bacteria present in a newborn's intestine (17). Gut microbiome of vaginally delivered children mainly consists of gram-negative bacteria capable of synthesizing lipopolysaccharides, which are known to be effective stimulators of innate immunity (20). In contrast, the microbiome of cesarean section (C-section) delivered babies is mainly composed of opportunistic pathogens circulating in the hospital's environment (21). These initial variations in microbial composition can strongly impact the innate lymphoid cell maturation. However, any defects in essential microbiota can be mitigated by immediate parent/caregiver-baby skin-on-skin contact or maternal vaginal/fecal microbiota transplantation to the baby, known as vaginal seeding (20–22). During lactation, breastfeeding provides specific prebiotics (such as inulin and human oligosaccharides) that stimulate the growth of commensal microbiota and thereby affect immune responses, particularly those of the innate arm (23). Eventually, the gut microbiome matures due to social and livestock interactions, infections and possibly active immunization via vaccination, resulting in an increased microbial diversity, which has a profound impact on both adaptive and mucosal immunity (17, 24).

In this review, we summarize the current knowledge on childhood leukemia risk factors that are known to influence the immune system and the microbial constitution of the child's gut. We discuss evidence on how factors such as childbirth mode, breastfeeding, commercial milk substitutes, early life social and livestock contacts, and vaccination may influence microbiome composition and increase or reduce the risk of ALL development. We also discuss how parents or caregivers can compensate for lack of microbial seeding in newborns after birth. Based on the collected evidence, we eventually provide simple recommendations that can be communicated to parents and caregivers to reduce the individual risk of childhood leukemia.

## 2 Fetal stage

### 2.1 Maternal nutrition and ALL risk

Robust evidence indicates that microbiome colonization already starts *in utero* (19, 25). Maternal diet influences fetal development by affecting epigenetic, DNA synthesis and repair processes. Furthermore, maternal diet affects fetal immune establishment and may potentially impact leukemia initiation (25). Maternal diet may influence the infant gut microbiome composition through vertical microbial transmission via vaginal delivery and breastfeeding, contributing to the infant's immune development (26). A study of mother-infant couplets recruited in the New Hampshire Birth Cohort investigating the association between maternal diet components and fetal microbiome confirms the influence of maternal diet on the infant gut microbiome as stratified by the delivery mode (26). The large multiethnic case-control California Childhood Leukemia Study (CCLS), concluded that higher maternal diet quality, rich in one-carbon nutrients and vitamin supplements before and during pregnancy correlated with a lower risk for ALL in offspring (OR = 0.88, Cl 0.78–0.98) and similarly a reduced risk for acute myeloid leukemia (AML) (OR = 0.76, Cl 0.52–1.11) (26). A recent meta-analysis on maternal diet and ALL risk indicated an inverse relationship between ALL risk and maternal consumption of fruits (OR 0.71; 95% Cl 0.59–0.86), as opposed to coffee intake (OR 1.45; 95%, Cl, 1.12–1.89) (27). This could be explained by the fact that fruits are a source of vitamins, minerals, and folate, all known to be implicated in DNA methylation and repair (25, 27) adjusted for maternal educational attainment and gestational diabetes, but not for socio-economic or maternal health status. In this study, adjustments were done for maternal educational attainment and gestational diabetes, but not for maternal health conditions or socioeconomic status. Similarly, several studies associate an elevated childhood leukemia risk to a diet low in vitamin A and minerals (particularly selenium) and report a direct association of reduced ALL risk and maternal diet containing eggs, seafood, fish and poultry meat, with the exception of red or processed meat (28–30). Eggs, fish and poultry are respectively rich in choline, folate and omega-3 fatty acids, which play an essential role in histone modification, anti-inflammatory processes and/or DNA methylation, reducing the likelihood of epigenetic alterations that may lead to chromosomal aberrations predisposing for ALL. Caffeine intake may inhibit DNA topoisomerase II, potentially giving rise to chromosomal aberrations described in childhood ALL (22, 31, 32). These findings apply to other food/drinks that contain DNA topoisomerase II inhibitors (Table 1). Moreover, studies within the NewGeneris cohort have examined maternal caffeine intake and an increased frequency of micronuclei in neonatal blood, linking dietary caffeine exposure to chromosome instability, genome rearrangements, and mutagenesis. However, chromosomal aberrations have not been analyzed directly.

**Table 1:** DNA topoisomerase II inhibitors present in food and environment (31).

**Substance**	**Source of exposure**
Benzene metabolites 4,4′-biphenol Catechol Hydroquinone p-benzoquinone	Gasoline stations Exhaust fumes
Catechins	Tea, wine, and chocolate
Dipyrone, baygon, thiram	Insecticides
Flavonoids Genistein Quercetin	Soy, legumes, apples, and berries
Senna	Anthraquinone laxative
Thiram	Agricultural laxative

Herbal tea intake in pregnancy seems to uphold protective properties against ALL occurrence, potentially due to its low caffeine content and richness in flavonoids engaged in anti-proliferative and antioxidant processes (33, 34). Tobacco and alcohol intake evidently remain a threat due to their interference respectively with caffeine and folate metabolism (25, 35).

Furthermore, dietary carcinogens including nitrosamines, ingested by the mother can pass through the placenta and expose the fetus to pre-leukemic transformative stimuli and increase the risk of childhood leukemia development (36). The NewGeneris Cohort study assessed the transplacental transmission of biomarkers of dietary exposure to carcinogens, such as oxidative fat metabolites, acrylamide, PAHs and nitrosamines (Table 2) in 1151 newborn cord blood samples. This was done by quantifying the levels of reactive metabolites bound to either hemoglobin or DNA (36). This technique primarily focuses on detecting adducts, which are formed when reactive metabolites bind to hemoglobin or DNA, as biomarkers of exposure to genotoxic compounds. Hemoglobin and DNA adducts were analyzed using high resolution mass spectrometry, whereas dioxin was measured via a commercially available validated bioassay (Dioxin Responsive Chemical Activated LUciferase gene eXpression or short “DR CALUX” bioassay). Indeed, most of the newborns were exposed to (pre)carcinogens and a successive analysis of this study found a significant association between the numbers of micronuclei in the cord blood lymphocytes and the level of exposure to maternal dietary carcinogens (36). Micronuclei are cytogenetic biomarkers whose frequency correlates to carcinogen-induced cancer risk. The study above highlights potential molecular mechanisms that may contribute to *in-utero* carcinogen-induced leukemia (36).

**Table 2:** Newborn exposure to carcinogens via maternal dietary intake during pregnancy.

**Carcinogen**	**Daily dietary maternal intake**	**Cord blood level**	**Source (food containing high levels)**
Oxidative fat metabolites (DNA adducts)	Omega 6 fatty acids 11.3 (1.2–77.0) g	34.2 (0.5–324.7) 10-9 nucleotides	Vegetable oils (soya, rape, and sunflower) Fatty meat Eggs
Acrylamide (hemoglobin adducts)	22 (1–135) μg	14.4 (4.4-124.8) pmol/g Hb	Coffee Crackers Biscuits Crisp bread Deep fried potato products (french fires, crisps)
Polycyclic aromatic hydrocarbons (DNA adducts)	80 (42–717) ng	8.4 (0.6–116.6) 10-8 nucleotides	Smoked and processed meat and/-or fish Barbecued/grilled meat
Nitrosamines (DNA adducts)	N-nitrosodimethylamine 82 (2–547)ng	0.40 (0.08–3.03) 10-8 nucleotides	Smoked meat Smoked fish Processed meat Preserved fish
Dioxin/PCBs (plasma)	77.2 (5.1–945.5) pg^†^	0.13 (0.01–104) pg/ml^†^	Full fat milk and dairy products Fatty meat Fatty fish

Data collected from NewGeneris database (36). ^†^2,3,7,8-tetrachlorodibenzo-p-dioxin (TCDD) toxic equivalency quotients.

Taken together, ALL is a multifactorial disease, whose development relies on an interplay between genetic and several environmental factors, including maternal exposure to dietary carcinogens. Current studies suggest that reducing exposure to dietary carcinogens could potentially mitigate genetic predispositions to ALL (Table 2). However, further research needs to be done to better understand the connection between maternal nutrition during pregnancy and the causative genetic factors. This should be analyzed in carefully controlled large mother-newborn cohorts, including for instance, dietary interventions. Investigating the impact of maternal antioxidant intake, or Mediterranean diet on ALL risk could provide valuable insights. In addition, mechanistic studies are necessary to clarify the role of maternal folate and omega-3 fatty acids in regulating DNA methylation and maintaining normal epigenetic marks in developing fetal cells.

## 3 Birth to infancy

### 3.1 Mode of delivery, prebiotics, vaginal seeding, skin-to-skin care, breastfeeding, and ALL risk

Mode of birth delivery represents a major contributor of microbiome colonization in newborns directly after birth (37). Vaginal delivery is considered as one of the most important early microbiome colonizing factors (38). This is supported by the fact that children delivered vaginally show a higher T-cell reactivity (lasting up to age of 2 years) and a more diverse gut microbiota composition compared to C-section delivered children (39). However, three large US case-control population-based studies of C-section delivery and childhood ALL reveal no strong association (40, 41). A similar finding was reported by the United Kingdom Childhood Cancer Study (UKCCS) (42). Other studies were able to identify a direct link between mode of delivery and overt childhood leukemia (38, 43). In a Californian registry-based case-control study, stratification of cases was done according to the major leukemia subtypes (38). Analysis of the correlation between C-section and childhood leukemia risk was based on a logistic regression model adjusted for accepted influencing leukemia risk factors such as breastfeeding, gestational age, household income and Hispanic ethnicity (38). Further stratified analyses revealed that a strong association exists C-section delivery and childhood ALL among Hispanic mother and child dyads (OR, 2.34; 95% CI, 1.23–4.46) (38).

It is commonly accepted that vaginal delivery accounts for a large mother-to-neonate microbial transmission (44). However, the conflicting evidence on C-section delivery and ALL risk makes a link between delivery-related microbiome alterations and ALL uncertain. Furthermore, the reduced share of maternal microbiome during C-section delivery can still be compensated for after birth by other mother-to-child microbial transmission routes like skin contact, breastfeeding or even prebiotic use and vaginal microbiome seeding (22, 23, 45). In terms of childhood leukemia prevention, inulin and human milk oligosaccharides (HMO) are considered as useful prebiotics due to their beneficial effect on stimulating the growth of benign commensal gut bacteria. Benign commensal gut bacteria reduce oxidative stress and decrease gut colonization by *Fusobacterium*, a bacterium known to display pro-cancer properties (23).

Vaginal seeding, a newly emerged concept, consists of mainly oral administration of vaginal fluid to newborns delivered via C-section, aiming to compensate for the lack of microbiome exchange in absence of a vaginal birth (46). This method of maternal bacteria transfer has been recommended by researchers due to a rising prevalence of C-section births (46). In the first study of vaginal seeding, published in 2016, Dominguez-Bello et al., showed that exposing C-section born children to their mother's vaginal fluid could enrich the gut microbiome similarly to the vaginally delivered counterparts (46). A more recent observational study including a larger number of newborns delivered via C-section mode that underwent vaginal seeding also reports a comparable microbiome composition between vaginally and C-section delivered babies (22). So far, there are no reported adverse side effects due to this intervention, including transmitted infections. However, as with vaginal deliveries, vaginal seeding carries the risk of hepatitis B virus, hepatitis C virus, HIV or herpes simplex virus (HSV) transmission (47). Considering these risks, we recommend more conventional alternatives of infant microbiome seeding such as skin-to-skin contact or breastfeeding.

The share of maternal microbiome to the child across six maternal and four infant body sites was calculated employing a fast-expectation maximization microbial source tracking algorithm (45). Breastfeeding was the predominant contributor of infant microbiome colonization (31.6%). However, a newborns' microbiota development benefitted from contact with other maternal sites including skin (25.7%), saliva (18.6%), nasopharynx (9.4%), feces (4.1%), and vagina (3.5%) (45). Importantly, 58.5% of infant microbiota can originate from any of the maternal transmission sources and the limited maternal microbial share due to a C-section delivery can easily be compensated (45).

Additional evidence attests to the essential role of skin-to-skin contact in building up a healthy newborn immunity by equipping the baby with beneficial microbes including *Staphylocococcus epidermis* which prevents potential pathogen colonization and exerts anti-inflammatory properties (48). Newborns experiencing skin-to-skin contact during the 1st h after birth revealed a greater share of maternal microbiota and consequently a more diverse microbiome compared to neonates without skin-to-skin contact (49). Taken together, immediate after birth skin-to-skin care, either with the infant's mother or a caregiver, is highly recommended (50). Additionally, several studies report a likelihood for the mother to initiate and carry on the breastfeeding practice after having experienced immediate skin-to-skin contact with their babies (51). That is associated with an elevated level of oxytocin, which is a vital lactation hormone that not only improves bonding and reduces stress levels, but also facilitates the process of milk release during breastfeeding (52).

Maternal antibodies, passively transferred to neonates through breastfeeding, provide a crucial protection against pathogens during early life (53). In a recent study it was found that antibody-mediated protective immunity can be obtained from the commensal microbiome of pregnant mice via breastfeeding (54). Studies examining breast milk composition discovered that puerperium-stage milk (< 1 week after birth) consists of ~70% immune cells which drop down to 0–2% in the postpartum period (>2 weeks after birth) (55, 56). Furthermore, profiling of mature-stage milk revealed three previously unknown and unique epithelial lactocyte subpopulations found to play a pivotal role in immune defense and intestinal development (55). Bogeart et al., described not only decreased microbial transmission resulting from the C-section births but, a more significant impact of breastmilk related to a higher amount of *Rothia mucilaginosa* presence found in the fecal microbiota of C-section born children (45). This bacterium has been shown to positively influence the gut microbiota by altering its composition. In mice studies, *R. mucilaginosa* increased the abundance of beneficial bacteria such as *Firmicutes* and *Lactobacillus* while reducing harmful bacteria like *Bacteroidetes* (57). These changes enhance gut health by promoting nutrient absorption and metabolic balance. This metabolic activity also produces short-chain fatty acids and other metabolites that have systemic effects, influencing gut health and maintaining immune balance by interacting with immune cells and cytokines (57). Preliminary studies suggest that some *R. mucilaginosa* exhibits anti-inflammatory properties. For instance, its abundance was found to be negatively correlated with pro-inflammatory markers such as interleukin IL-8 and IL-1β in a cohort of adults with bronchiectasis (58). This may help in modulating inflammation by interacting with Toll-like receptors (TLRs) and other immune pathways (58). In addition, the findings from Bogeart et al., reiterate that breastfeeding may partially make up for the reduced infant microbial seeding upon C-section delivery and breastmilk represents the biggest contributor to newborn gut microbiome (31.6% of microbial content) compared to other after-birth transmission routes (45). Large meta-analysis studies from 2015 and 2021 suggest that continuation of breastfeeding for at least 6 months may result in a decrease of childhood leukemia incidence by 14–20% (59). However, there is noticeable differences in childhood leukemia rates and breastfeeding practices between high-income (HIC) and medium-low income countries with lower breastfeeding rates in HIC. Clearly, maternal socioeconomic status influences the choice to breastfeed and its duration. Aiming to rule out the socioeconomic differences, an analysis was carried out focusing only on 12 studies conducted in the HIC countries. The result unveiled a statistically significant inverse correlation between childhood leukemia and breastfeeding for over 6 months (OR, 0.84; 95% CI, 0.78–0.91) (59). Breastfeeding offers a low-cost and usually accessible public health measure for childhood leukemia prevention. However, breastfeeding practice faces many barriers, including lactation problems, infant behavior, early return to work, socioeconomic status, lack of social support and self-efficacy, and unsupportive childcare (60). Hence, for the mothers unable to breastfeed, immediate skin-to-skin contact, early life social, and livestock contact, vaccination, as well as daycare attendance provide alternative and effective stimuli for an early priming of the infant's immunity.

It is calculated that infants receive a load of up to 1 million immune cells in every feeding (61). Human milk, in comparison to commercial sources, consists of maternal immune cells and prebiotics (e.g., HMOs) (56). For instance, HMOs offer an essential protection in the context of an immature immunity and represent the third largest component of breastmilk. By contrast, commercial milk only contains traces of this complex sugar (61). For over a decade, market statistics show a drastic increase of babies fed with commercial milk formula instead of human milk (62, 63). A three-paper series published in 2023 in Lancet raises the concern of challenged breastfeeding practice due to highly predatory tactics used by formula milk industry (64). In one of the series, they outline the long-term benefits of breastfeeding in fighting disease for both mothers and the newborns by providing an immune boost that cannot be reproduced by commercial milk substitutes (65). Examining the relationship between exclusive breast milk and pure milk powder in preventing leukemia, a large retrospective case-control study of children diagnosed with leukemia vs. healthy controls found that the consumption of commercial milk powder instead of breastmilk might significantly increase the incidence of overt childhood leukemia (66). Whereas, analysis of the association between duration of breastfeeding and childhood leukemia incidence predictably indicated a slightly reduced leukemia risk upon >6 months of breastfeeding (67). Although a better understanding of the biological mechanisms between breastfeeding and risk of childhood leukemia is needed, existing data indicates a protective effect of breastmilk against leukemia development.

## 4 Early childhood

### 4.1 Social and/or livestock contacts and ALL risk

Early life microbial colonization is essential for the maturation of immune system and originates from the maternal microbiota (37). Although, microbial colonization might commence *in utero*, it is a persistent natural process and its largest share happens after birth. Hence, daycare attendance could help maintain a nurtured microbiome and facilitate immune system maturation through early exposure to common infections, which in turn may reduce the risk of ALL development (68). In a Danish childcare database study the ALL risk for children attending childcare is estimated to be reduced by 32% (69). However, these findings are not supported by a second recent Danish cohort study in which, childcare attendance shows no significant reduction in the context of ALL risk reduction (68). Since the enrolment age was 2–14 years in the later study, it could be inferred that immune modulation following microbiome alterations is critical to ALL risk during the 1st years of life (68).

The “delayed infection” theory postulates that early infections decrease the risk of childhood ALL. Early infections have shown to be beneficial in priming the immune system (3). Epidemiological studies on exposure to infectious agents and immune challenges by proxy in infancy (< 1 year of age) support a protective effect of early infections against ALL (3). Taking this into account, we suggest early life livestock contacts, siblingship, or daycare attendance as they may provide the necessary after-birth microbiome colonization and subsequently protection against ALL.

In line with the “delayed infection” theory, a small ALL cluster was identified in Milan among seven children following AH1N1 swine flu (12). No evidence of previous exposure to ionizing and non-ionizing radiation or other leukemic causative chemicals was uncovered. Since none of the children attended nursery during the 1st year of life and six out of seven were firstborn, it is probable that lack of exposure to infections during early life became the reason of these ALL cases (12, 70). Interestingly, a German nationwide, population-based assessment of the influence of COVID-19 pandemic on the incidence of BCP-ALL childhood cancers revealed a general increase in 2020, which dropped below average in 2021 (71).

Additionally, a large-scale epidemiological study conducted after Germany's reunification showed a 25% higher childhood ALL incidence in the former East Germany as compared to only 1% increase in West Germany and the rest of Europe, over the same period of 6 years (72). In contrast to West Germany, nearly all the babies in the eastern part had to attend state nursery centers, a practice which was discontinued after reunification (72). Hence, cessation of universal daycare attendance after reunification may in part account for this shift (72). Further supportive evidence on this stems from a recent meta-analysis of 7,847 leukemia cases and 11,667 controls by the Childhood Leukemia International Consortium (73). The consortium demonstrated that regular contact of children (< 1 year of age) with livestock, poultry and pets reduced the risk of ALL development significantly (livestock: OR = 0.65, 95% CI: 0.50, 0.85) (73).

### 4.2 Vaccination and ALL risk

Acquiring infections during the 1st year of life strengthens the immune system due to antibody production (74). “Trained immunity” during early life resulting from vaccination is a newly emerging concept which could impact childhood leukemia prevention as it primes the immune system and boosts immunity (75). For instance, the Bacille Calmette-Guerin (BCG) vaccination is thought to shape the innate immune response by production of trained natural killer cells (NK), macrophages and monocytes (76). So far, many epidemiological meta-analyses investigating the association between early life vaccination and ALL risk have been carried out, but only BCG vaccination has shown a protective effect in terms of childhood ALL prevention (74, 76). In a retrospective study, R. Rosenthal was the first to describe the role of BCG vaccination in childhood ALL incidence declension in 1972 (77). Since then, other observational studies suggest the valuable effect of BCG vaccination in reducing leukemia incidence (75, 78). However, no firm conclusion can be made as the outcome of these studies were often inconsistent. A lack of assessment of other ALL influencing factors including environmental stimuli (microbes/etiological agents), social contacts or mother-to-child interaction may account for the variable outcome. Notably, countries with an active BCG vaccination policy report the lowest childhood leukemia incidence (74, 77). The protective association between BCG vaccine and childhood leukemia rate was also observed while comparing ALL cases among children living in either East or West Germany before and after the reunification. In the former East, opposite to West Germany, BCG vaccination was mandatory (in addition to daycare attendance), until reunification (76). Accordingly, data showed a lower childhood leukemia incidence in the former East Germany with 3/100,000 children as compared to 3.7/100,000 in former West Germany, but reunification eventually canceled out this disparity (76). The influence of different vaccines, such as rubella, measles, mumps, diphtheria-tetanus-pertussis (DTaP) poliomyelitis, hepatitis B (HBV), and BCG in affecting the incidence of childhood ALL has also been explored in a large meta-analysis study (74). Results showed no evidence of reduced leukemia risk for all the other vaccines, whereas analysis of early vaccination (< 3 months of age) with the BCG vaccine revealed a statistically robust protection from ALL (74). These associations are supported by numerous studies reporting on BCG vaccination of newborns and leukemia incidence in Austria, Chicago, Quebec and Germany (74).

The hypothalamus-pituitary-adrenal axis hypothesis by Schmiegelow proposes that early life infections lead to an increase in plasma cortisol levels and may consequently facilitate the elimination of preleukemic cells (69). Vaccine administration can also increase plasma cortisol, which might account for how vaccination may prevent childhood ALL development (79). Additionally, in line with Greaves' delayed infection theory, exposure to vaccines' pathogen-associated molecular patterns (PAMPs) stimulates innate immune response, which could mimic common infections acquired due to nursery attendance (74). However, further research is necessary to elucidate this concept, as this does not explain why reduction of leukemia risk appears to be limited to only the BCG vaccine.

## 5 Discussion and recommendations for the target population

**Figure 4:**
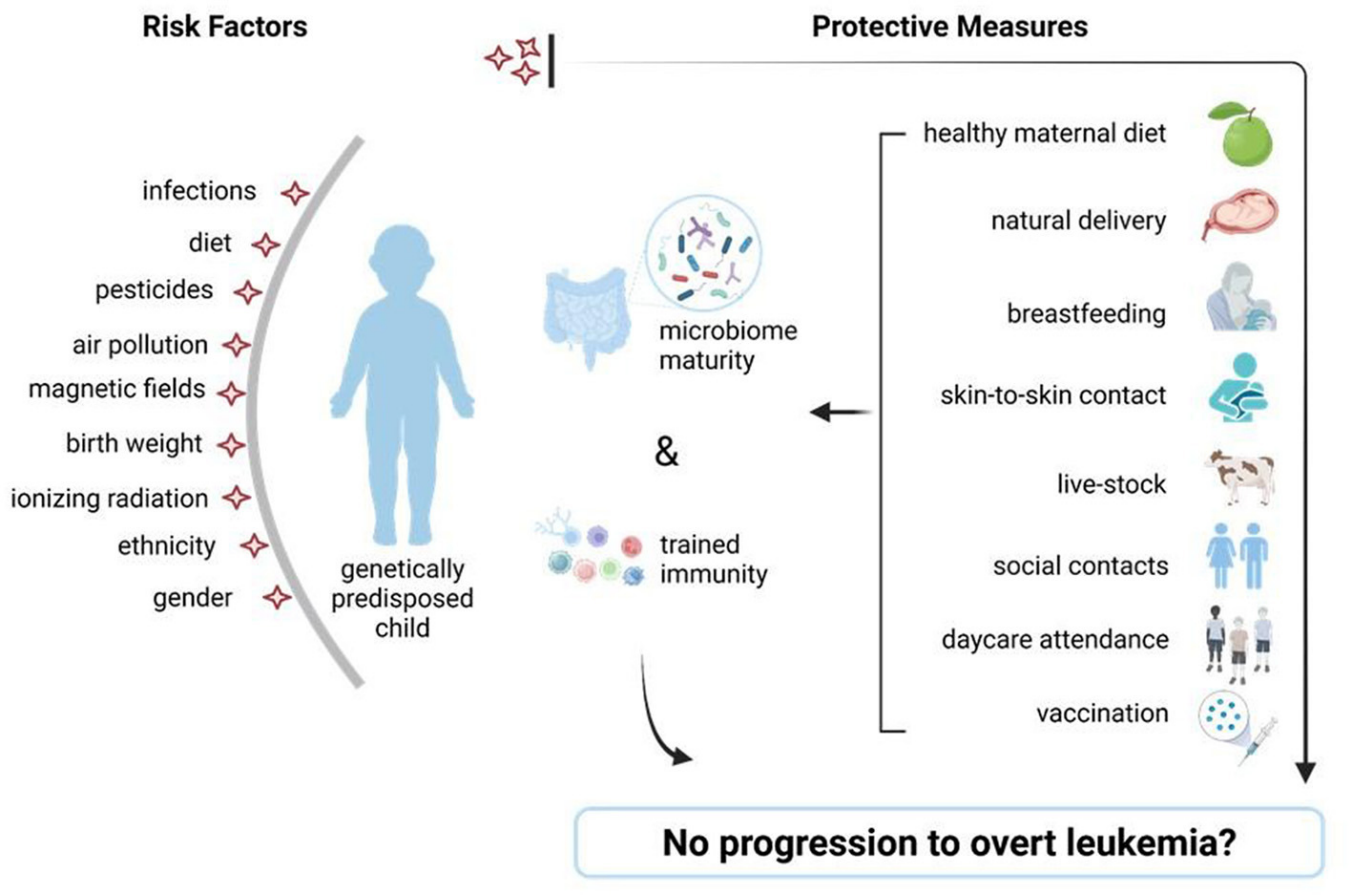
Risk factors for childhood leukemia and potential protective measures. Schematic view of accepted childhood leukemia risk factors **(left)** and preventive measures **(right)** which assure intestinal diversity and a mature immunity, able to prevent the switch from preleukemia toward overt leukemia, in genetically predisposed children. Figure was created on BioRender.com.

Microbiome-targeted interventions are an emerging area of research for preventing ALL in genetically predisposed children and several prevention strategies, including diet modulation and/or early-life microbial exposure are currently under investigation. Epidemiological and experimental studies demonstrate that a healthy gut microbiome holds great potential in protecting against childhood leukemia development (2, 10, 23, 25). Several environmental factors (prenatal and especially postnatal) reviewed above may provide the adequate gut microbiome diversity that could aid in the protection against ALL development through the establishment of strong early life immunity (Figure 4) (4, 17, 20, 23, 24, 46, 69, 72, 80).

As reported by several epidemiological and experimental studies, diet during pregnancy may strongly influence maternal and fetal microbiome and provide the bacteria needed for “priming” of the offspring's immunity (25, 26, 29, 30).

C-section births have generally increased over the past two decades, potentially reducing microbial exchange at birth. While interventions like vaginal/fecal microbiome seeding remain still debatable, we strongly advocate early skin-to-skin mother-to-infant/and, or guardian-to-infant contact to promote microbial transfer and subsequent early immune priming, as supported by epidemiological studies. Early skin-to-skin contact is an important determinant in shaping a strong immunity. Therefore, an uninterrupted skin-to-skin mother or caregiver-infant contact for at least the 1st hour after birth, particularly for the non-vaginally born babies is recommended. Similarly, early social and livestock contact may offer an additional strategy toward childhood leukemia prevention (45, 46, 48–50, 72, 73).

Bogaert et al., demonstrates that breastmilk is the major source of newborn microbiota and can greatly compensate on its own for the lack of maternal microbial transmission after a C-section delivery. Hence, if feasible for the mother both physically and mentally, we encourage this practice as well as the implementation of breastfeeding-friendly measures at the workplace and focus on maternal mental wellbeing (45, 55, 56, 59).

Additionally, epidemiological studies show that vaccination and early exposure to infections or pathogens have a protective role against childhood leukemia, as well as asthma and allergies. Hence, overtly sterile environments and limited interaction with siblings or pets may serve as a trigger for dysregulated immune responses later on and negatively influence the onset of leukemia among genetically predisposed children (74–77, 79).

To advance these prevention strategies (dietary adjustments and early-life microbial exposure) further studies are needed to clarify the mechanisms linking gut microbiome alterations to ALL. For families with a genetic predisposition, adopting a healthy diet, avoiding unnecessary antibiotic use, and exploring evidence-based probiotic therapies could be practical steps to support microbiome health and potentially reduce ALL risk (1, 10, 11, 23, 80, 81). However, consulting healthcare professionals before starting any intervention is essential to ensure safety and efficacy.

## 6 Conclusions

**Figure 5:**
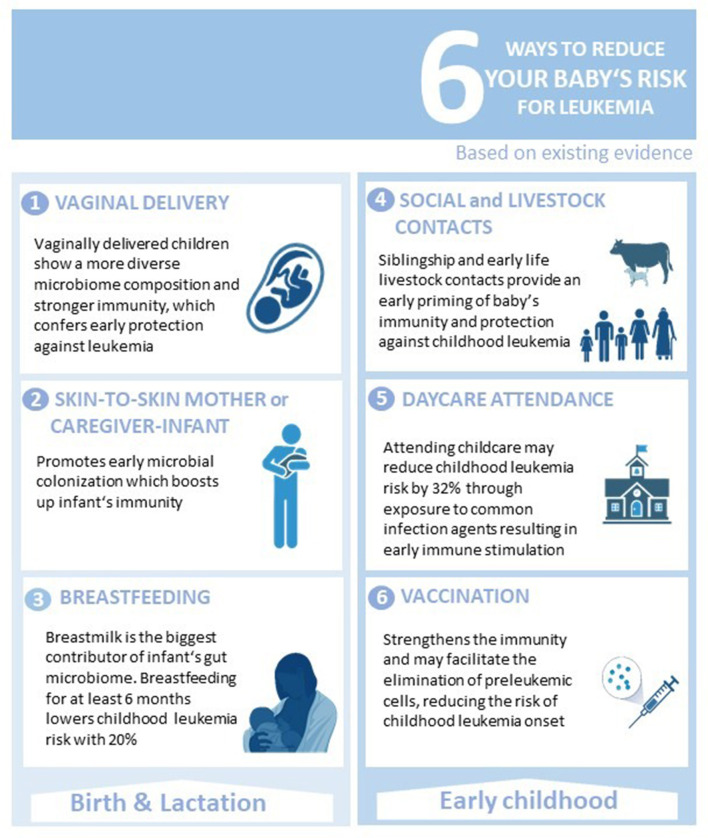
Simple measures toward childhood ALL prevention. Six ways parents and/or other caregivers can contribute in reducing the risk of childhood ALL development. Figure was created on BioRender.com.

Growing evidence suggests that early childhood ALL is in principle a preventable disease and the key to its prevention is nurturing early life gut microbiome and immune maturation that can be achieved effectively and safely through a healthy maternal diet, breastfeeding, vaginal mode of delivery, early skin-to-skin mother/caregiver-to-infant contact, nursery attendance, vaccination, and early life social and livestock contacts to facilitate exposure to common infection agents (Figure 5).
